# Anchorage and Bond Strength of SBPDN Bar Embedded in High-Strength Grout Mortar

**DOI:** 10.3390/ma19010002

**Published:** 2025-12-19

**Authors:** Takaaki Itoh, Ryoya Ueda, Bunka Son, Ayami Kuno, Yuping Sun

**Affiliations:** 1 Toyota T&S Construction Co., Ltd., 65 Kamimukaida, Kamekubi-cho, Toyota 470-0375, Japan; ito1841@toyota-ts.co.jp (T.I.); kuno2528@toyota-ts.co.jp (A.K.); 2 Shimizu Corporation Co., Ltd., 2-16-1 Kyobashi, Chuo-ku, Tokyo 103-8370, Japan; r_ueda@shimz.co.jp; 3 Graduate School of Engineering, Mie University, 1577 Kurimamachiya-cho, Tsu 514-8507, Japan; 4 Graduate School of Engineering, Kobe University, 1-1 Rokkodai-cho, Nada, Kobe 657-8501, Japan

**Keywords:** anchorage, bond strength, pull-out resistance, sheath duct, grout mortar

## Abstract

The SBPDN (Steel Bar Prestressed Deformed Normal relaxation) bar, which has ultra-high yield strength yet much lower bond resistance than conventional deformed bars, has been recently proposed to be used as the longitudinal rebar instead of a normal-strength deformed bar to simply realize strong earthquake-resilient concrete components. To facilitate and promote the application of concrete components reinforced with SBPDN rebars to the structures located in earthquake-prone regions, it is indispensable to develop reliable and effective anchoring means and clarify the bond strength of SBPDN bars embedded in concrete and/or grout mortar. This paper presents experimental information on the pull-out tests of fifteen SBPDN bars embedded in grout mortar, along with a discussion on the effective anchorage details and the bond strength of SBPDN bars. The tested SBPDN bars have a nominal diameter of 22.2 mm, the maximum diameter currently available on the market. All SBPDN bars were embedded in high-strength grout mortar with a targeted compressive strength of 60 MPa. The primary experimental variables included the end anchorage details, the diameter of sheath ducts, and the embedded length of the bars. Test results demonstrated that either screwing two nuts and a washer at the end of SBPDN bars or providing a rolling-threaded end region was effective in preventing them from premature slip from grout mortar. If the embedment length was 20 times the bar diameter or longer, the proposed two anchorages could ensure the SBPDN bar to fully develop its specific yielding strength as high as 1275 MPa. In addition, it has also been experimentally revealed that the bond strength of SBPDN bars embedded in grout mortar was much lower than that of conventional deformed bars and varied between 2.84 MPa and 3.98 MPa.

## 1. Introduction

Since the 1995 Southern Hyogo Prefecture earthquake, which was one of the most severe earthquakes to affect a modern urban area and cause catastrophic disaster, human societies have witnessed serious damage and numerous losses of life induced by several mega-earthquakes with higher seismic intensities than anticipated in modern seismic design codes [1,2,3,4,5,6]. In addition to causing serious damage to buildings and infrastructures, these recent mega-earthquakes [7,8,9,10,11,12,13] also presented a new challenge to the structural engineering community. The damage observed in concrete buildings during recent mega-earthquakes has demonstrated that ductile concrete structures conforming to current design codes and/or standards could survive strong ground motion without collapsing but might be left with such severe damage and residual deformation that the buildings would be hard to repair after these earthquakes. From these lessons [7,8,9,10,11,12,13] and the perspectives of quick re-occupancy of facilities and prompt resumption of social activities, the structural engineering community has recently recognized the importance of resilience and post-earthquake restorability for concrete structures and made extensive efforts to develop resilient concrete components and/or structure systems [14,15,16,17,18,19,20,21,22,23,24,25].

As of now, there are two methods proposed to make resilient concrete components and/or structures. One involves the utilization of the unbonded prestressed tendon (UPT) to clamp the precast concrete wall panels to the foundation and make a so-called rocking wall system. The other makes use of weakly bonded ultra-high strength (WBUHS) bars as longitudinal rebars of concrete components, including columns and walls.

The rocking wall systems were initiated by Priestley et al. [14,15], and the static and dynamic responses of the rocking walls have been extensively studied by many researchers [16,17,18,19,20,21]. The last author of this paper and his colleagues have proposed using SBPDN rebars in lieu of conventional deformed rebars to simply realize resilient concrete components and experimentally verified the high seismic resilience of cast-in-site and/or precast concrete columns and walls reinforced by SBPDN rebars [22,23,24].

**Figure 1:**
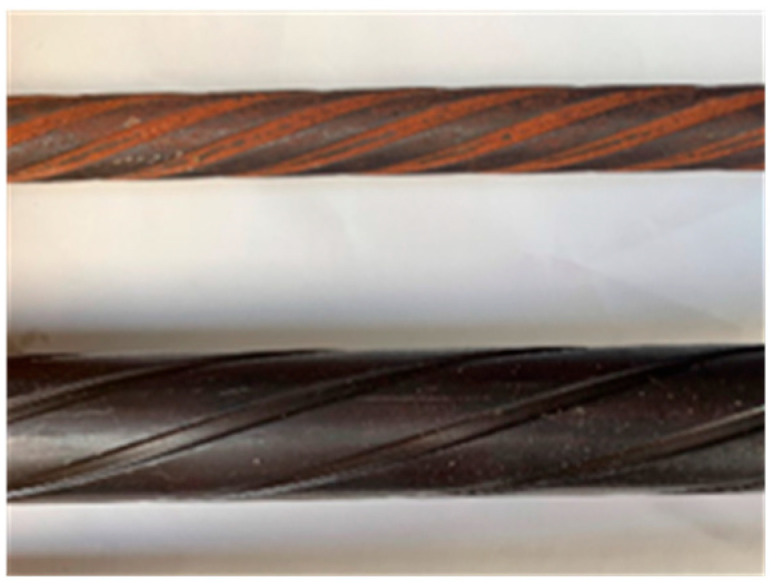
Schematic view of SBPDN bars (The upper is U12.6 bar, and the lower is U22.3 bar).

SBPDN bars have been made for the prestressed concrete structures. As shown in Figure 1, an SBPDN bar has a spiraled or helical groove on its surface, which significantly weakens its bond resistance, though the SBPDN bar has a specific yield strength as high as 1275 MPa. According to Funato et al. [25], when embedded in concrete with a compressive strength of 42 MPa, the bond strength of an SBPDN bar was about 3.0 MPa, which is only one-fifth or one-sixth of that of a deformed bar. Interestingly, it is the low bond strength that mitigates the development of the axial stress induced by combined axial compression and seismic lateral force, delays the yielding of longitudinal rebars, and leads to a stable increase in lateral resistance sustained by them up to large deformation, providing concrete columns and walls with high resilience [22,23,24]. If not being reliably anchored, however, the low bond strength tends to cause large premature slip before the SBPDN bar develops its high yield strength, reducing the resistance by longitudinal SBPDN rebars and making them not follow the plane-remain-plane assumption, which is an indispensable assumption when evaluating the ultimate capacities of concrete components.

Therefore, to promote the application of concrete components reinforced by SBPDN rebars into practice, it is of great importance to develop reliable and effective anchoring means to avoid excessive slip of SBPDN rebars before fully developing high yield strength. Meanwhile, to rationally and accurately evaluate the seismic capacity of cast-in-site and/or precast concrete components utilizing SBPDN rebars, information on the bond strength between the SBPDN bar and concrete and/or grout mortar is also desirable.

Extensive studies have been conducted on the bond stress–slip relationship between concrete and deformed bar, which has much higher bond strength than SBPDN bar, as summarized in references [26,27,28,29]. The previous studies [26,27,28,29] primarily dealt with the bond strength and necessary embedment length for the deformed bars embedded into cast-in-site beam–column joints. While there are several studies on the bond features between high-strength PC wire strand and grout mortar [30,31,32,33], these previous studies concentrated on the evaluation of the bond strength of high-strength PC wire strand embedded in the prestressed and precast concrete girders. To the authors’ best knowledge, there is neither information on the bond behavior between the SBPDN bar and grout mortar nor development of the reliable anchorage methods for the SBPDN bar embedded in grout mortar for the precast resilient concrete components.

The headed bars have been developed and used to reduce congestion of concrete in the beam–column joint. In light of the experimental studies [34,35,36], ACI 318-19 [4] and AIJ 2024 [6] recommended Equations (1) and (2), respectively, to determine the embedment length (L_e_) of headed rebars anchored into a concrete beam–column joint. Due to the limited data on the behavior of headed bars, particularly of the high-strength bars and concrete, in ACI 318-19 [4] and AIJ 2024 [6], design provisions for the embedment length limit the yield strength of headed reinforcing bars to 414 MPa and 490 MPa, respectively.

(1) $$\frac{L_{e}}{d_{b}}=\frac{f_{y}\sqrt{d_{b}}}{31\sqrt{f_{c}}},\;f_{y}\leq 414\;MPa$$

(2) $$\frac{L_{e}}{d_{b}}=\frac{0.7f_{y}}{10\times \left(0.9+f_{c}/40\right)},f_{y}\leq 490\;MPa$$

where f_y_ and f_c_ express the yield strength of the bar and the concrete strength, respectively, and d_b_ represents the nominal diameter of the bar.

**Figure 2:**
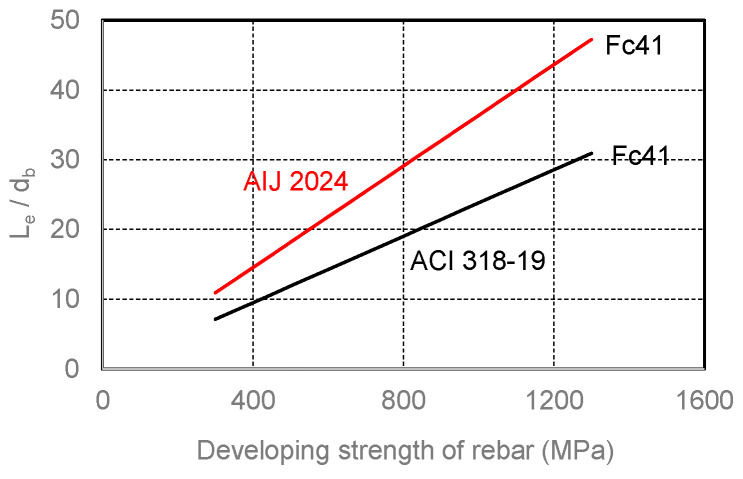
Examples of embedment length versus developing strength relationships of headed bars [4,6].

Figure 2 compares the prescribed embedment lengths of headed rebars in the form of the developing strength of the steel bar versus the embedment length relationship. The solid red and black lines represent the calculated results by Equations (1) and (2) for a headed bar (22 mm in diameter) embedded in concrete with a compressive strength of 41.4 MPa, which is the upper limit of concrete strength prescribed in ACI 318-19.

As apparent from Figure 2, using normal-strength (f_y_ < 414 MPa) headed rebars, the necessary embedment length can be significantly reduced. ACI 318-19 gives a shorter necessary embedment length than AIJ 2024 [6]. However, for higher-strength rebars than 414 MPa and/or 490 MPa, the embedment length will increase linearly along with the developing strength. For an SBPDN rebar to develop its high yield strength (1275 MPa), the embedment lengths will reach 24.4 times (ACI 318-19) or 49.9 times (AIJ 2024 [6]) the bar diameter, the latter of which is obviously not realistic. Therefore, more effective and realistic anchorage methods need to be developed from the perspective of facilitating the application of SBPDN rebars.

The objectives of this paper are as follows: (1) to propose reliable anchoring means for the SBPDN bar embedded in grout mortar; (2) to verify the effectiveness of the proposed anchorage methods and find the minimum embedment length; (3) to clarify the influence of the diameter of sheath ducts on the bond resistance of SBPDN bars; and (4) to present experimental data on the average bond strength of SBPDN bars embedded in grout mortar, contributing to the future revision of current codes.

## 2. Experimental Program

### 2.1. Outline of the Proposed Anchorage Methods

**Figure 3:**
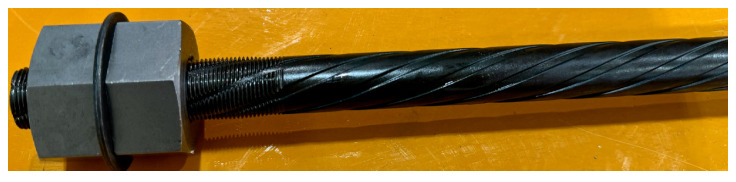
Appearance of the proposed anchorage by nuts and a washer (A-method).

To address the difficulty inherent in effectively anchoring high-strength rebars, this paper proposes two simple yet effective anchorage methods for SBPDN rebars. One of the proposed anchoring means consists of two nuts and a washer (A-method) as shown in Figure 3, and the other is to provide a rolling-threaded region at the end of bars (S-method). As shown in Figure 3, in the A-method, two nuts and a washer are screwed onto the rolling-threaded end of the SBPDN bar to prevent it from premature slip and to be reliably anchored.

### 2.2. Outlines of Specimens

To verify the effectiveness of the proposed anchorage methods, a total of fifteen SBPDN bars (U22.2) with a nominal diameter of 22.2 mm, which is the maximum diameter available on the market, were tested under monotonic pull-out loading. The experimental variables were the embedment length (15 d_b_, 20 d_b_, and 25 d_b_), the anchorage detailing (A: nuts and washer; S: rolling threaded end; N: null), and the diameter (55 mm, 65 mm, and 100 mm) of sheath ducts, all of which had an identical thickness of 0.32 mm.

**Figure 4:**
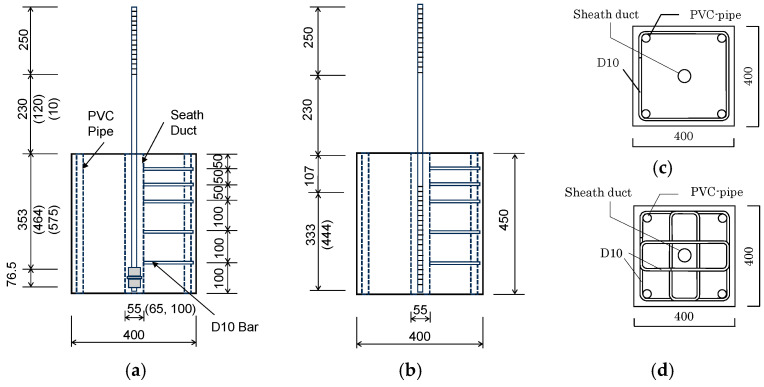
Dimensions and reinforcement details: (**a**) specimens anchored by nuts and washers; (**b**) specimens with rolling threaded ends; (**c**) S1 cross-section; (**d**) S2 cross-section.

Figure 4 illustrates dimensions of specimens and reinforcement details of the concrete block where SBPDN bars were embedded. The concrete blocks had a 400 mm square cross-section and a height of 450 mm, 560 mm, and 670 mm for the specimens with the embedment length of 15 d_b_, 20 d_b_, and 25 d_b_, respectively. The numerals in the parentheses in Figure 4 are shown for the specimens with 20 d_b_ and 25 d_b_ embedment lengths. Table 1 lists experimental properties of all specimens.

To avoid premature splitting failure of the concrete block, D10 deformed bars were used to confine the concrete block, and two types of configurations were used for the D10 hoops (see Figure 4c,d). All specimens were divided into two groups (S1 and S2) according to the configuration type of D10 hoops and the testing date.

In the notations of specimens listed in Table 1, the first alphabet expresses the anchorage means (A: nuts and washer; S: rolling threaded end; N: null), the next combination of numeral and alphabet (e.g., 15 d) represents the embedment length (15 times the diameter of the bar), the second next combination of alphabet and numeral (e.g., T55) expresses the inner diameter of the sheath duct (55 mm), and the last combination of alphabet and numerals (S1 or S2) represents the configuration type of D10 hoops in the concrete block. The S1 and S2 groups of specimens were tested in 2021 and in 2022, respectively.

It is noted that in the specimens with embedment lengths of 20 d_b_ and 25 d_b_, the spacing of the three sets of D10 hoops near the top of the concrete block was enlarged from 50 mm to 75 mm, while the spacing of the D10 hoops placed in the lower part of the concrete block was maintained at 100 mm. In addition, the top 20 mm portion of each SBPDN bar embedded in grout mortar was unbonded by overlaying the surface of the bar with clay and then wrapping the end 20 mm region with insulating tape.

Of the fifteen specimens, the SBPDN bars in eleven A-specimens were anchored at their ends by “nuts and washers”, primarily aiming at clarification of the minimum embedment length. The anchorage of the SBPDN bar in the two S-specimens was provided by the rolling-threaded end region of the bars with two different lengths of 15 d_b_ and 20 d_b_. In the two N-specimens, there was no special anchorage means provided at the end of the SBPDN bar to investigate the average bond strength of the SBPDN bars.

The mechanical properties of SBPDN bars and the D10 deformed bar are listed in Table 2. Each value in Table 2 represents the average of three test coupons.

Ready-mixed concrete with targeted strengths of 30 MPa was used to make concrete blocks. Portland cement and coarse aggregates with a maximum particle size of 20 mm were used to make the concrete. Three standard concrete cylinders (100 mm in diameter and 200 mm in height) were prepared for the specimens tested on the same day, and all concrete cylinders were cured under the same conditions as the specimens. The actual compressive cylinder strengths measured at the testing stages are given in Table 1.

Non-shrink grouting cement was used to make grout mortar with a W/C ratio of 0.18.

The compressive strengths of grout mortar measured at the testing date for each specimen are also shown in Table 1.

**Figure 5:**
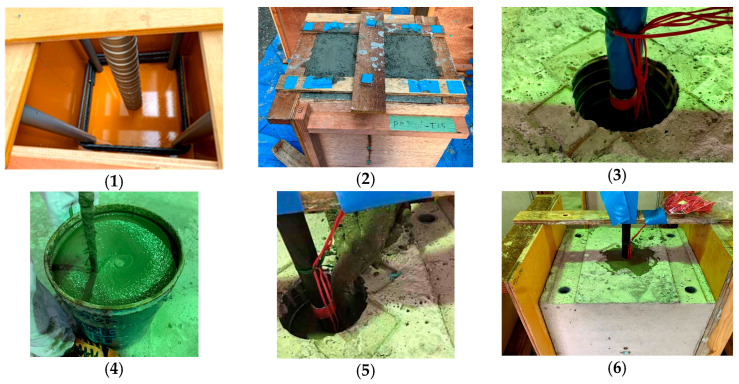
Procedures for making specimens: (**1**) fixing sheath duct; (**2**) casting concrete; (**3**) positioning SBPDN bar; (**4**) mixing grout; (**5**) injecting grout; (**6**) curing on air.

The procedures of making specimens are summarized in Figure 5 and described as follows: (1) putting steel cages into formworks and fixing sheath ducts; (2) casting concrete; (3) calibrating the position of SBPDN bars; (4) mixing the grout mortar; (5) injecting or casting grout mortar into the sheath duct; and (6) curing the grout mortar on air.

### 2.3. Loading Apparatus and Instrumentations

**Figure 6:**
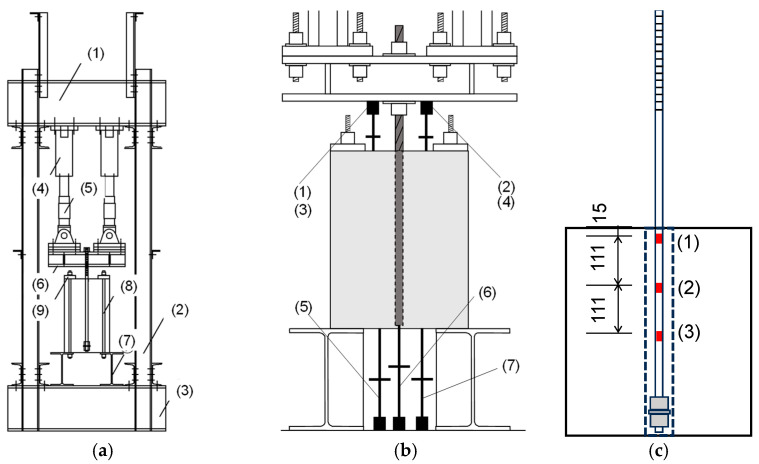
Loading apparatus and instrumentation: (**a**) loading frame; (**b**) positions of displacement transducers; (**c**) examples of the locations of strain gages.

Figure 6 illustrates the loading apparatus along with the positions of displacement transducers and examples of the locations of strain gages attached to SBPDN bars. As shown in Figure 6a, after fixing the concrete block to the loading frame (1)–(3) by fastening four PC bars (8) and (9) passing through PVC pipes embedded at the corners of concrete block (see Figure 4), monotonic pull-out loading was applied via two 300 kN hydraulic jacks, (4) and (5), that were fixed to the reaction steel frame. The upper end of the SBPDN bar was fixed to a stiff W-shaped steel beam (6) by nuts. The lower ends of four PC bars were anchored to two W-shaped steel beams (7).

Seven displacement transducers (DTs) were used to measure the upper and lower displacements of the SBPDN bar, as shown in Figure 6b. The distance (L_u_) from the top surface of the concrete block to the lower end of the upper loading W-shaped steel beam (7) varied between 46 mm and 291 mm. The average of the displacements measured by four DTs (1)–(4) is used as the top displacement (D_t_), while the bottom push-in displacement (D_b_) or the end slip (S_b_) of the SBPDN bar can be obtained by Equation (3).

(3) $$D_{b}=D_{6}-\frac{D_{5}+D_{7}}{2}=S_{b}$$

It is noted that only two DTs were attached to measure the upper displacement of the specimens with an embedment length of 25 d_b_ because the clear distance (L_u_) was too short to accommodate more displacement transducers.

Strain gages were attached to the surface of the portion of the SBPDN bars embedded in grout mortar, except for the two S-specimens, because their lower end regions were rolling threaded. As shown in Figure 6c, three gages (1)–(3) were attached at an interval of 111 mm onto SBPDN bars with L_e_ = 15 d_b_, and four and five gages were attached to measure the strains of the SBPDN bars with L_e_ = 20 d_b_ and 25 d_b_, respectively.

## 3. Test Results and Discussion

### 3.1. Primary Test Results and Observed Behavior

Table 3 shows primary test results concerning the pull-out load-carrying capacities of SBPDN bars. P_y_ given in Table 3 represents the yielding load calculated by multiplying the yield strength (see Table 2) by the nominal cross-sectional area (387 mm^2^) of SBPDN bars.

**Figure 7:**
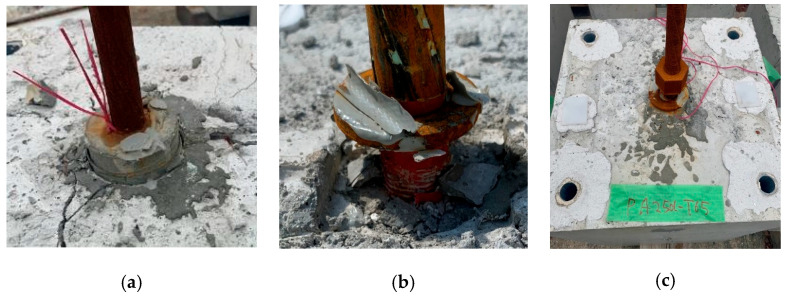
Observed ultimate or failure modes: (**a**) pull-out of sheath duct (PSD); (**b**) pull-out of SBPDN bar (PB); (**c**) yielding of SBPDN bar (PY).

Figure 7 illustrates the three ultimate or failure modes observed during the pull-out tests. They are the pull-out of the sheath duct (PSD), the pull-out of the SBPDN bar (PB), and the yielding of the SBPDN bar (YB). The PSD mode means that the sheath duct was pulled out from the concrete block along with injected grout mortar and the SBPDN bar before reaching the yielding load P_y_ due to insufficient anchorage. The PB mode represents that only the SBPDN bar was pulled out from the injected grout mortar at much lower loads than P_y_ due to a substantial lack of bond resistance of the SBPDN bars. The PY mode is the desirable mode, in which the SBPDN bar end could be reliably anchored and develop its ultra-high yielding strength.

As apparent from Table 3, five of the SBPDN bars with the embedment length of 15 *d_b_* exhibited PSD mode, implying that even when anchored by nuts and washers, the embedment length of 15 d_b_ could hardly provide the SBPDN bars with sufficiently reliable anchorage to develop their yield strength. Only specimen A15d-T65-S2 reached the yield load P_y_. Comparing the results of two pairs of specimens with identical configurations of transverse reinforcement in concrete blocks (A15d-T65-S1 and A15d-T100-S1, A15d-T55-S2 and A15d-T65-S2), one can see that increasing the diameter of the sheath duct housing the SBPDN bar could increase the pull-out resistance of the SBPDN bar, but more effective confinement of the concrete block was indispensable for the SBPDN bar to fully develop its high yield strength when the embedment length was 15 d_b_. Meanwhile, it is evident that the rolling-threaded end region with L_e_ = 15 d_b_ (specimen S15d-T55-S2) could not ensure enough anchorage to the SBPDN bar either. In addition, one can also see from Table 3 that the ratio of the measured pull-out capacity to the yield strength had a mean value of 1.020, a standard deviation of 0.0051, and a coefficient of variance of 0.0049, respectively, which implies that the sheath duct diameter had little, if any, influence on the ultimate pull-out capacity of SBPDN bars with an embedded length of 20 d_b_ or longer.

**Figure 8:**
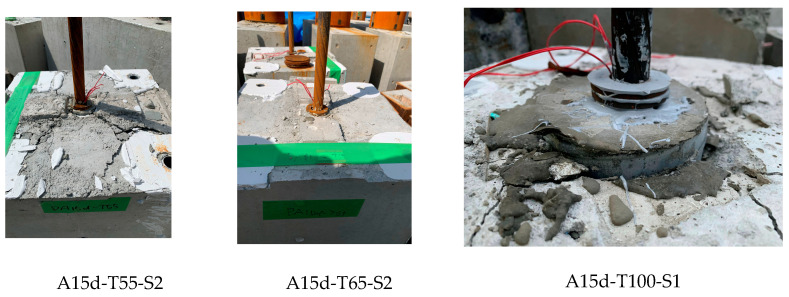
Examples of surface cracks observed in specimens with L_e_ = 15 d_b._

No splitting failure or surface cracks were observed in the specimens with L_e_ = 20 d_b_ or longer, while some surface cracks appeared ultimately on the top surfaces of specimens with L_e_ = 15 d_b_. Figure 8 shows examples of surface cracks observed at the top of the concrete block for the specimens with L_e_ = 15 d_b_. It can be seen from Figure 8 that no surface cracks appeared in specimen A15d-T65-S2, which developed the yield strength due to more effective confinement by overlapped hoops.

The primary test results shown in Table 3 further indicate that extending the embedment length to 20 d_b_ or longer, the proposed end anchorage (by nuts and washers, A-method) could ensure reliable and firm anchorage to the SBPDN bar to develop its ultra-high yield strength. Providing a rolling-threaded end region (S-Method) with L_e_ = 20 d_b_ (specimen S20d-T55-S2) was also able to ensure the SBPDN bar developed its yield strength. On the other hand, if without any anchorage means provided at the ends, the pull-out resistance of the SBPDN bar (specimen N20d-T55-S2) was very small, even though the embedment length was extended to 20 d_b_.

It is noted that the ultimate mode of specimen A25d-T100-S1 was judged as indeterminate. The pull-out loading was prematurely terminated before reaching the yield load P_y_ (524 kN) because the hydraulic jacks were out of tune after reaching about 494 kN.

### 3.2. Pull-Out Load Versus Upper Pull-Out Displacement Relationships

**Figure 9:**
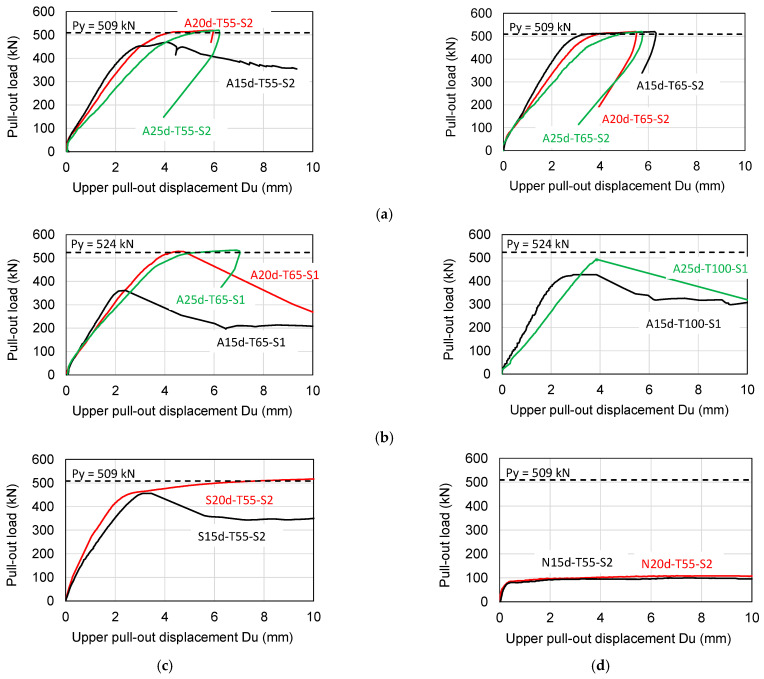
Pull-out load versus upper pull-out displacement relationships: (**a**) A-specimens with overlapped hoops; (**b**) A-specimens with a single hoop; (**c**) S-specimens; (**d**) N-specimens.

The measured pull-out load (P) versus upper pull-out displacement (D_u_) relationships are displayed in Figure 9. For the specimens that developed the yield load-carrying capacity (P_y_), the pull-out load was unloaded a while after reaching P_y_ for the sake of the safety of the loading apparatus. The black, red, and green solid lines shown in Figure 8 represent the test results of SBPDN bars with embedment lengths of 15 d_b_, 20 d_b,_ and 25 d_b_, respectively. The horizontal dotted lines superimposed in each graph of Figure 9 express the calculated yield load P_y_. The upper pull-out displacement is obtained by subtracting the elastic elongation from the top displacement (D_t_) in the form of Equation (4).

(4) $$D_{u}=D_{t}-\frac{PL_{u}}{EA}$$

where P is the measured pull-out load, L_u_ is the distance from the top surface of the concrete block to the bottom face of the upper loading W-shaped steel beam ((6) in Figure 6a), and E and A are the Young’s modulus (see Table 2) and the cross-sectional area (387 mm^2^) of the SBPDN bar, respectively.

As apparent from Figure 9a,b, screwing nuts and washers at the end of the SBPDN bars ensured reliable and sufficient anchorage for them to fully develop the yield strength if the embedment length was 20 d_b_ or longer. For the A-specimens with L_u_ = 15 d_b_, by comparing the measured P-D_u_ curves of A15d-T55-S1 and A15d-T100-S1 with that of specimen A15d-T65-S2, one can see that the larger the diameter of the sheath duct, the higher the pull-out resistance of the SBPDN bar, but confinement by a single hoop could not ensure sufficiently firm anchorage even when the diameter of the sheath duct was enlarged to 100 mm. On the other hand, if confining a concrete block with overlapped hoops, the embedment length of 15 d_b_ might provide anchorage strong enough for the SBPDN bar to develop its yield strength. It is noted that the longer the embedment length, the larger the upper pull-out displacement during the early loading stage until the SBPDN bar reaches the P_y_. This is because the upper pull-out displacement shown in Figure 9 included the elastic elongation of the portion of the SBPDN bar embedded in grout mortar.

It can also be seen from Figure 9c that the rolling-threaded end with a length of 20 d_b_ and confinement by overlapped hoops (specimen S20d-T55-S2) could also enable the SBPDN bar to develop the yield strength because of the stronger confinement by overlapped hoops than by a single hoop [37,38].

Figure 9d obviously indicates that just extending the embedment length could not increase the pull-out resistance of SBPDN bars if no anchorage means were provided at the end of the bar. The pull-out load increased nonlinearly along with the pull-out displacement until D_u_ reached about 0.4 mm, but from then on, it maintained nearly constant, accompanied by an abrupt increase in the pull-out displacement or slip of SBPDN bars from the grout mortar (see Figure 7b).

### 3.3. Histories of the Axial Strains of SBPDN Bars

**Figure 10:**
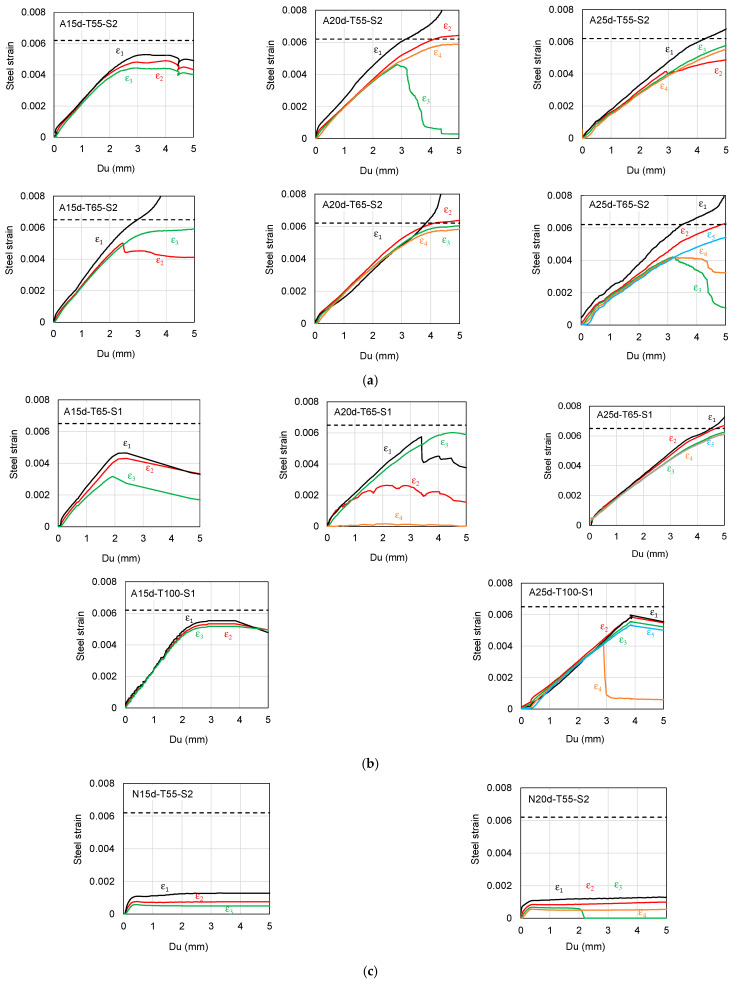
Measured histories of the steel strains of the SBPDN bars: (**a**) A-specimens with overlapped hoops; (**b**) A-specimens with a single hoop; (**c**) N-specimens.

To better see how the proposed anchorage methods affected the pull-out resistant behavior of SBPDN bars, Figure 10 shows the histories of steel strains of SBPDN bars measured by the strain gages (1)–(3), (1)–(4), and/or (1)–(5), respectively, for the bars with embedment lengths of 15 d_b_, 20 d_b_, and 25 d_b_.

The five solid lines with different colors and labeled as ε_1_ through ε_5_ represent the measured strains from the top end downward to the lower portion embedded in grout mortar. The horizontal black dotted lines superimposed in Figure 10 express the yield strains of SBPDN bars. No steel strains of SBPDN bars are plotted in Figure 10 for the S-specimens because it was impossible to attach strain gages on the surface of the rolling threaded portion embedded in grout mortar (see Figure 4b).

As is obvious from Figure 10, the strain ε_1_ exhibited a larger value than the others because it represents the strain measured at the location outside the top end of the embedded portion and not subjected to the influence of surrounding concrete and/or grout mortar like the others. For the SBPDN bars embedded in concrete blocks confined by overlapped hoops (A-S2 specimens), the shorter the embedment length, the greater the steel strains of SBPDN bars developed at the smaller upper pull-out displacement (D_u_). This can be attributed to the fact that the longer embedded portion lowered the required axial strain to render the same D_u_ as the shorter embedded portion caused.

An important observation can be made from Figure 10a that the measured strains (ε_2_ through ε_5_) of SBPDN bars embedded within grout mortar developed at nearly identical rates along with D_u_ until the strains reached 0.004, regardless of the difference in embedment length. The little difference among these steel strains demonstrates that the contribution to the pull-out action by the bond between SBPDN bars and grout mortar was very little and that the end anchorage by nuts and washers worked as expected and effectively resisted the pull-out action until the tensile strain and stress developed in SBPDN bars reached 0.004 and 800 MPa or higher, respectively. Beyond that, strain and stress levels, some differences, and divergence appeared among strains ε_2_ through ε_5_. Since some strain gages broke due to the bar movement and weakness of mortar at large displacement, it is not judicious to use the measured strains to evaluate the bond resisting properties at large displacement for SBPDN bars anchored by the A-method. The divergency and distribution of the steel strains observed at large pull-out displacement do not change the force transfer mechanism between the bar and mortar because the steady increment of strain ε_1_ indicated that the primary resistance to the pull-out action was rather provided by the anchorage of nuts and washers than by the bond action between the bar and mortar.

For the SBPDN bars embedded in grout mortar confined by a single hoop, the measured strains exhibited nearly the same increasing tendency as the strain ε_1_ was up to the strain of 0.002 (see Figure 10b). After that strain, some difference was observed among the measured strains of SBPDN bars with a 65 mm sheath duct and an embedment length of 15 d_b_. This strain difference, however, tended to decrease as the embedment length increased. One can see from Figure 10b that either lengthening the embedment length to 20 d_b_ or increasing the diameter of the sheath duct to 100 mm, the measured strains of SBPDN bars within grout mortar showed little difference from the strain ε_1_ until the strain of 0.004. These observations indicate that in A-specimens, if the embedment length was 20 d_b_ or longer, nuts and washers screwed at the ends of SBPDN bars provide dominant resistance to the pulling-out loading or action. It is noteworthy from Figure 10b that even enlarging the diameter of the sheath duct to 100 mm, the SBPDN bar with an embedment length of 15 d_b_ could not effectively develop its yield strain due to slippage of the grout from the sheath duct.

If no anchorage means were provided at the ends of SBPDN bars, the steel strains measured within the grout mortar exhibited a different increasing rate from the early stage of the pull-out loading, as shown in Figure 10c. The strain gradient between two adjacent measured strains remained nearly constant along with the pull-out displacement, and the maximum measured steel strain was much lower than the yield strain (0.0062 or 0.0065). This fact means that the bond between the SBPDN bar and grout mortar primarily resisted the pull-out action but could not provide sufficient resistance for the SBPDN bars to avoid excessive slip at much lower load-carrying capacities than the yield load.

**Figure 11:**
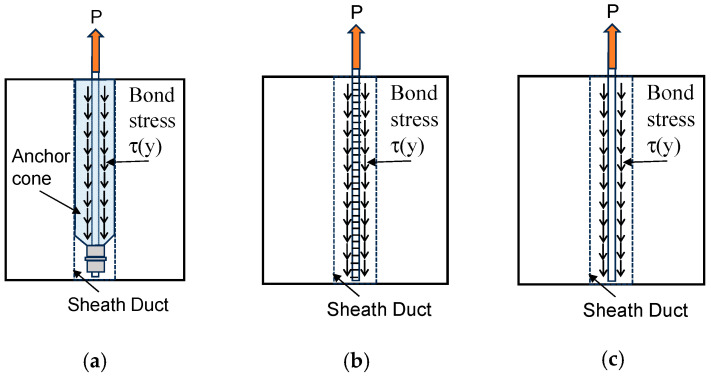
Idealization of the pull-out resisting mechanisms of SBPDN bars with different anchorage: (**a**) A-method; (**b**) S-method; (**c**) no anchoring means.

Based on the above-described observations, the main pull-out resistance mechanisms of SBPDN bars embedded in grout mortar are summarized in Figure 11. As shown in Figure 11, in A-specimens, the nuts and washers worked like the head of an anchor and played a dominant role in resisting the pull-out action, but the contribution by the bond stress between the bar and mortar was little. On the other hand, in S-specimens and N-specimens, the pull-out action was resisted only by the bond stress.

### 3.4. Bond Stress–Slip Behavior and Bond Strength of SBPDN Bars Embedded in Grout Mortar

To investigate the bond stress–slip behavior of SBPDN bars embedded in grout mortar, only the measured steel strains of specimens N15d-T55-S2 and N20d-T55-S2 will be used because they do not involve the influence of the end anchorage means.

**Figure 12:**
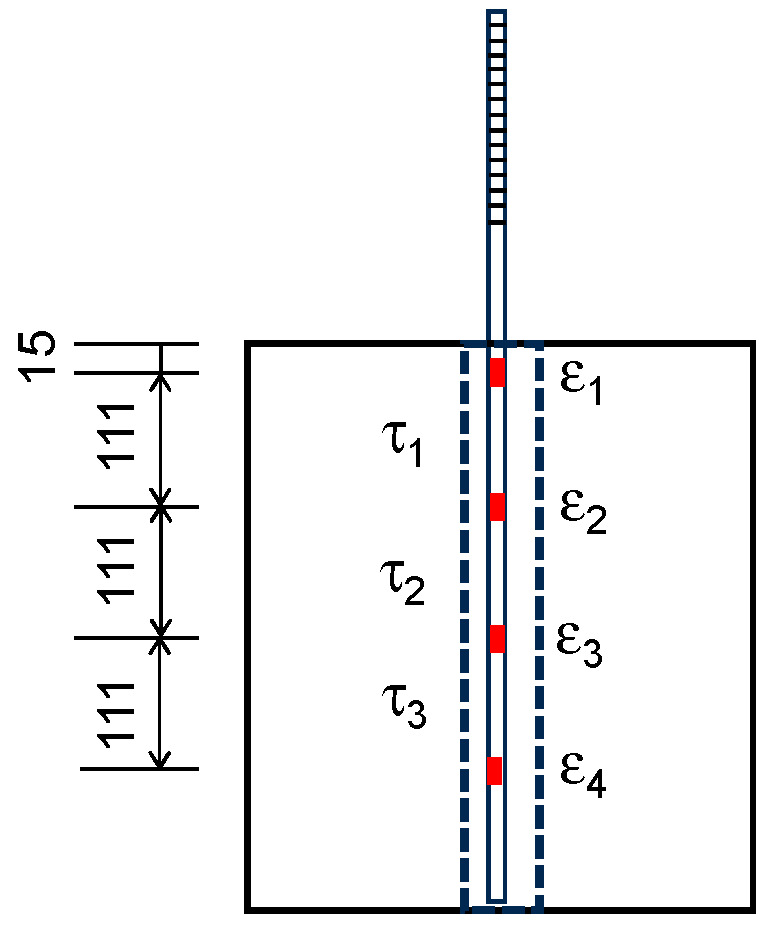
Locations of strain gages and segmental bond stress.

Figure 12 displays the locations of strain gages as well as segmental bond stress (τ_i_) between any two adjacent strain gauges. The average of the upper pull-out displacement (D_u_) and the lower end one (D_b_) can be taken as the average slip (S). Equations (5) and (6) define the local bond stress (τ_i_) and the average bond strength (τ_ave_) over the embedment length (L_e_), respectively.

(5) $$\tau_{i}=\frac{\left(\epsilon_{i}-\epsilon_{i+1}\right)EA}{\pi d_{b}L_{i}}(i=1,2,\ldots ,n-1)$$

(6) $$\tau_{ave}=\frac{P}{\pi \times d_{b}\times L_{e}}$$

where ε_i_ is the measured strain of the i-*th* strain gage, and L_i_ is the distance between two adjacent strain gages (111 mm). As can be seen from Figure 10c, the steel strains measured within the sheath ducts of the two N-specimens were much less than the yield strain (0.65%, see Table 2) of the SBPDN bar, implying that the linear elastic stress–strain relation inherent in Equation (5) is reasonable and acceptable.

**Figure 13:**
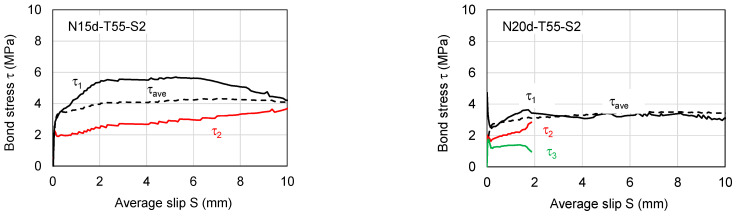
Measured bond stress–slip relationships of N-specimens.

Figure 13 shows the measured bond stress versus average slip relationships of N-specimens. For comparison, the average bond stress (τ_ave_) versus slip curves are also plotted in Figure 13. The bond stresses τ_2_ and τ_3_ in specimen N20d-T55-S2 are terminated near the average slip of 2.0 mm because the strain gages defining them broke at that slip.

As apparent from Figure 13, the segmental bond stress near the upper end of grout mortar or concrete block exhibited the largest value. The longer the embedment length, the smaller the segmental and average bond stress. This observation, however, does not mean that the bond strength of the SBPDN bar decreases along the embedment length because the strain ε_1_ measured the strain outside the embedded portion of the SBPDN bar and hence does not necessarily reflect the real bond resistance between the bar and grout mortar. It can also be seen from Figure 13 that the bond stress τ_2_ of specimen S15d-T55-S2 and that of specimen S20d-T55-S2 exhibited nearly the same varying tendency with the slip until 2.0 mm, and the segmental bond strength varied between 2.84 MPa and 3.98 MPa.

**Figure 14:**
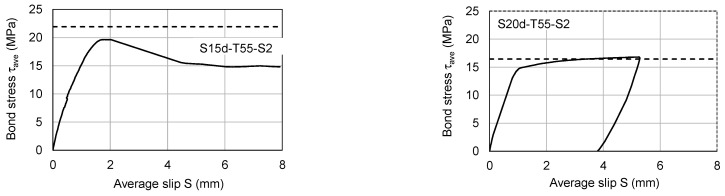
Measured average bond stress–slip relationships of S-specimens.

To better understand how the rolling-threaded end influenced the pull-out resistance of SBPDN bars, Figure 14 shows the measured bond stress–slip relationships averaged over the embedment length of the two S-specimens. The horizontal dotted lines shown in each graph of Figure 14 represent the calculated bond stress (τ_y_) necessary for the SBPDN bar to develop its yield strength (1316 MPa, see Table 2).

It is obvious from Figure 14 that the average bond stress gradually and nonlinearly increased along with the slip until it approached near 15.0 MPa with little, if any, difference between the two S-specimens. Beyond 15.0 MPa, the measured bond stress in specimen S15d-T55-S2 increased at a faster rate than that of specimen S1d5-T55-S2. This observation, however, does not mean that the longer the embedment length, the smaller the bond strength of the rolling-threaded SBPDN bar, because the bond stress of 15 MPa in specimen S15d-T55-S2 reached about 92% of the calculated τ_y_ (16.5 MPa) and exceeded it at larger slip. On the other hand, even the maximum bond stress (19.6 MPa) of specimen S15d-T55-S2 was only 89% of the calculated τ_y_ (21.9 MPa). Those observations imply that 15.0 MPa may approximately give a lower limit of bond strength to the rolling-threaded SBPDN bars and that the minimum embedment length shall be 20 d_b_ to provide firm anchorage for SBPDN bars embedded in grout mortar to develop the yield strength.

## 4. Conclusions

To develop an effective and reliable anchorage method for SBPDN rebars adopted in drift-hardening concrete components, this paper investigated the pull-out resistant behavior of large-size SBPDN bars with a nominal diameter of 22.2 mm. A total of fifteen SBPDN bars were tested under monotonic pull-out loading. The SBPDN bars were embedded in high-strength grout mortar housed in a sheath duct and concrete block. The primary experimental variables were the end anchorage detailing, the embedment length of SBPDN bars, and the diameter of the sheath duct. Based on the experimental research described in this paper, the following conclusions can be drawn:

1. Screwing two nuts to clamp one washer at the end of SBPDN bars (A-method) was observed to be able to provide SBPDN bars with sufficiently firm anchorage, enabling them to develop the ultra-high strength yield strength if the embedment length was twenty times the nominal diameter or longer. One A-specimen (A25d-T100-S1) was judged as indeterminate just because the jacks were out of tune before the pull-out load reached the yield load. The embedment length of 20 d_b_ is much shorter than those (24.4 d_b_ and 49.6 d_b_) obtained by extrapolating ACI 318-19 and AIJ 2024 [6] provisions, respectively. For headed high-strength deformed bars and facilitates the application of SBPDN rebars to actual concrete components.
2. Providing a rolling-threaded end region (S-method) was also observed to be able to ensure SBPDN bars fully develop the yield strength when the embedment length was twenty times the nominal diameter or longer. The lower limit of the average bond strength of the rolling-threaded SBPDN bars was observed to be about 15.0 MPa.
3. Diameter of sheath duct did not significantly influence the ultimate pull-out resistant capacity of SBPDN bars embedded in grout mortar but did mitigate the requirement of contribution to the pull-out action provided by the bond between the bar and grout mortar. The larger diameter of the sheath duct seemed to increase the pull-out resistance by the proposed end anchorage A- and S- methods. Furthermore, the simplicity of the proposed A- and S-methods obviously could facilitate the injection of grout mortar and improve detailing in the joint regions of precast concrete components reinforced by SBPDN rebars.
4. The SBPDN bar itself exhibited much lower bond strength than deformed bars, and the measured bond strength of 22.2 mm SBPDN bars varied between 2.84 MPa and 3.98 MPa. Therefore, to straightly anchor SBPDN bars into the beam–column joints or the foundation beams is not a feasible means because it would cause large premature slip, hinder the development of steel stress, and degrade the load-carrying capacity of concrete components reinforced by SBPDN rebars.

The above are the main findings drawn from the study described in this paper. While each test data represented the result of a single specimen due to the limit of the budget, the variance of the ultimate pull-out capacities of A- and S-specimens that reached the yield strength was very small. Therefore, the proposed anchorage means can be expected to contribute to future revision of existing code provisions.

## Figures and Tables

**Table 1:** Experimental properties of specimens.

Specimen	*f_c_^′^* [MPa]	*f_g_^′^* [MPa]	Embedment Length (mm)	Anchor Type	Diameter of Sheath Duct (mm)	Configuration Type of Hoops
A15d-T55-S2	39.6	72.2	15 d_b_ (333)	A	55	Overlapped
A15d-T65-S1	36.0	66.3			65	single
A15d-T65-S2	39.6	72.2			65	Overlapped
A15d-T100-S1	33.3	65.1			100	single
S15d-T55-S2	39.6	74.5		S	55	Overlapped
N15d-T55-S2	39.6	74.5		N	55	Overlapped
A20d-T55-S2	38.3	74.8	20 d_b_ (444)	A	55	Overlapped
A20d-T65-S1	33.3	65.1			65	single
A20d-T65-S2	38.0	74.2			65	Overlapped
S20d-T55-S2	38.3	74.8		S	55	Overlapped
N20d-T55-S2	38.0	74.2		N	55	Overlapped
A25d-T55-S2	40.7	74.2	25 d_b_ (555)	A	55	Overlapped
A25d-T65-S1	36.2	75.5			65	single
A25d-T65-S2	40.7	74.2			65	Overlapped
A25d-T100-S1	37.5	75.5			100	single

*f_c_^’^*: concrete cylinder (100 × 200 mm) strength; *f_g_^’^*: grout mortar cylinder (50 × 100 mm) strength.

**Table 2:** Mechanical properties of steel used.

Notation		Grade	$E_{s}$ [GPa]	$f_{sy}$ [MPa]	$\varepsilon_{y}$ [%]	$f_{su}$ [MPa]
D10		SD295A	167.8	344	0.20	436
U22.2	S1 Specimens	SBPDN1275	212.0	1355	0.65	1479
	S2 Specimens		211.1	1316	0.62	1455

*E_s_*: Young’s modulus; *f_sy_* and *ε_y_*: yield strength and strain; *f_su_*: tensile strength.

**Table 3:** Primary test results.

Specimen	*P_max_* [kN]	*P_y_* [kN]	*P_max_*/*P_y_*	Ultimate or Failure Mode
A15d-T55-S2	468	509	0.920	PSD
A15d-T65-S1	361	524	0.690	PSD
A15d-T65-S2	520	509	1.022	PY
A15d-T100-S1	427	524	0.815	PSD
S15d-T55-S2	456	509	0.896	PSD
N15d-T55-S2	100	509	0.197	PB
A20d-T55-S2	520	509	1.022	PY
A20d-T65-S1	528	524	1.008	PY
A20d-T65-S2	520	509	1.022	PY
S20d-T55-S2	520	509	1.022	PY
N20d-T55-S2	109	509	0.214	PB
A25d-T55-S2	520	509	1.022	PY
A25d-T65-S1	534	524	1.019	PY
A25d-T65-S2	521	509	1.024	PY
A25d-T100-S1	494	524	0.943	Indeterminate

## Data Availability

The original contributions presented in this study are included in the article. Further inquiries can be directed to the corresponding authors.
